# A combined oro-nasopharyngeal swab is more sensitive than mouthwash in detecting SARS-CoV-2 by a high-throughput PCR assay

**DOI:** 10.1007/s15010-021-01600-1

**Published:** 2021-03-18

**Authors:** Wiebke Michel, Jacqueline Färber, Milica Dilas, Hans-Gert Heuft, Ina Tammer, Jannik Baar, Achim J. Kaasch

**Affiliations:** 1 Medical Faculty, Institute of Medical Microbiology and Hospital Hygiene, Otto-von-Guericke-University Magdeburg, Leipziger Str. 44, 39120 Magdeburg, Germany; 2 Institute of Transfusion Medicine and Immunohematology, Otto-von-Guericke University Hospital, Leipziger Strasse 44, 39120 Magdeburg, Germany

**Keywords:** Diagnostic accuracy, Sensitivity, Specificity, COVID19, SARS2, Mouthwash, Oronasopharyngeal swab

## Abstract

**Objectives:**

The optimal diagnostic specimen to detect SARS-CoV-2 by PCR in the upper respiratory tract is unclear. Mouthwash fluid has been reported as an alternative to nasopharyngeal and oropharyngeal swabs. We compared mouthwash fluid with a combined oro-nasopharyngeal swab regarding test performance.

**Methods:**

In a large refugee facility, we retested individuals with a previous positive test for SARS-CoV-2 and their quarantined close contacts. All individuals were asymptomatic at the time of testing. First, a mouthwash (gargling for at least 5 s) with sterile water was performed. Then, with a single flocked swab the back of the throat and subsequently the nasopharynx were sampled. Samples were inactivated and analysed on a Roche cobas 6800^®^ system with the Roche SARS-CoV-2 test.

**Results:**

Of 76 individuals, 39 (51%) tested positive for SARS-CoV-2 by oro-nasopharyngeal swab. Mouthwash detected 13 of 76 (17%) infections, but did not detect any additional infection. Samples that were positive in both tests, had lower cycle threshold (*Ct*)-values for oro-nasopharyngeal samples, indicating a higher virus concentration, compared to samples only positive in oro-nasopharyngeal swabs.

**Conclusion:**

Mouthwash is not as sensitive as combined oro-nasopharyngeal swab in detecting upper respiratory tract infection.

## Introduction

In December 2019, a new lung disease called Coronavirus Disease 2019 (COVID-19) first appeared in Wuhan, China, and subsequently spread globally [1]. The causative agent is the severe acute respiratory syndrome coronavirus 2 (SARS-CoV-2). SARS-CoV-2 is an enveloped, single-stranded positive-sense RNA virus. Together with SARS-1 and MERS coronavirus it is classified in the *Orthocoronaviridae* subfamily, genus *Betacoronavirus* [2].

SARS-CoV-2 is efficiently transmitted from person to person by respiratory droplets [3]. Rapid and accurate detection of the virus is essential to contain outbreaks. One recommended diagnostic specimen for SARS-CoV-2 detection is the nasopharyngeal swab [4], but combined naso-oropharyngeal swabs can increase the sensitivity of SARS-CoV-2 detection [5]. A meta-analysis of different SARS-CoV-2 studies showed the highest detection rates in sputum, followed by nasopharyngeal and then oropharyngeal swab samples [6]. In severe cases of COVID-19 or at later stages in the disease, SARS-CoV-2 can be detected in samples from the lower respiratory tract, such as sputum or bronchial aspirate [7].

Expected shortages of swabs and the unpleasant experience of a nose swab as reported during an outbreak in a refugee facility led us to assess alternative diagnostic specimens. Although, available evidence at that time suggested that saliva was a suitable specimen [8], we decided against using saliva for two reasons: first, our main automated PCR system is sensitive to viscous samples such as saliva; and second, saliva may not be produced in sufficient quantity by all subjects. Therefore, we decided to test mouthwash as a specimen. To rigorously assess test performance, we compared mouthwash and a combined oro-nasopharyngeal swab in a study population with expected low viral loads.

## Methods

The study was conducted in a large refugee facility on two occasions in May 2020 during an outbreak in the facility. Residents (age > 6 years) of the facility that were previously tested positive for SARS-CoV-2 (*n* = 63) and their quarantined close contacts (family members, roommates and friends, *n* = 13) were prospectively enrolled in the study. Participants were retested for SARS-CoV-2 with mouthwash and a combined oro-nasopharyngeal swab in controlled conditions. Each individual entered the study only once with the first positive sample. Samples were taken by previously instructed medical personnel. A single flocked swab (eSwab™ Copan) was used to sample the back of the throat and subsequently the deep nasopharynx. For the mouthwash, residents were instructed to gargle the mouth with 10 ml sterile water for at least 5 s under the supervision of medical personnel. All residents were interviewed for any symptoms in the preceding three weeks that were compatible with a SARS-CoV-2 infection using a questionnaire with the following items: fever, tiredness, dry cough, sneezing, body ache, nasal congestion, sore throat, diarrhoea, headache, shortness of breath, loss of smell or taste, or any other symptoms. Interviews and sample collection took place with the assistance of interpreters; gargling was demonstrated, if necessary.

Samples were transported at room temperature and stored overnight at 4 °C. All samples were mixed 1:1 with ATL buffer and analysed with the cobas^®^ SARS-CoV-2 assay on the Roche cobas 6800 system according to the manufacturer's instructions. Detection of the *E*-(envelope)-gene and *Orf1/a* (open reading frame 1) or only *E*-gene or only *Orf1/a* were interpreted as confirmation of SARS-CoV-2 infection. Cycle threshold (*Ct*)-values above 40 were considered as negative.

## Results

Overall, 76 individuals were tested. Of these, 63 individuals had a previous positive PCR-test for SARS-CoV-2 at a median time of 14 days prior and 13 individuals were in quarantine as close contacts. Among the quarantined close contacts, five new infections were detected in a second round of testing, whereas seven did not contract the infection. Age ranged from 7 to 59 years, with an average age of 29 years (Table 1). At the time of testing, no individual showed any symptoms of COVID-19. 26 individuals recollected symptoms compatible with COVID-19 in the past three weeks (Table 1): headache (12 individuals), cough (10), rhinitis (9), loss of taste (6), fatigue (5), mild shortness of breath (4), aching limbs (4), sore throat (3), fever (3), diarrhoea (2), and sneezing (2). In some of the individuals, the symptoms lasted only one day. None was hospitalised.

**Table 1:** Overview of the study population stratified by age

Category	Number of individuals by age group (in years)						
	< 10 *N* = 1	10–19 *N* = 11	20–29 *N* = 37	30–39 *N* = 14	40–49 *N* = 7	50–59 *N* = 6	Total *N* = 76
Male/female	0/1	9/11	30/7	12/7	5/2	5/1	61/15
Positive PCR-test preceding study and positive test at time of study	0	8	17	5	2	2	34
Positive PCR-test preceding study, but negative test at time of study	0	1	16	7	2	3	29
Negative PCR-test preceding study, but positive test at time of study	0	2	1	1	1	0	5
Negative PCR-test preceding study and negative test at time of study	1	0	3	1	2	1	8
Individuals with symptoms male/female	0/0	5/0	12/3	3/1	1/0	1/0	22/4
Symptoms (male/female)							
Aching limbs			3/0	1/0			4/0
Cough			7/2			1/0	8/2
Diarrhoea		1/0		1/0			2/0
Fatigue			3/1	1/0			4/1
Fever			3/0				3/0
Headache		1/0	6/2	2/0	1/0		10/2
Loss of smell or taste			5/0	1/0			6/0
Rhinitis		2/0	5/1		1/0		8/1
Mild shortness of breath			3/1				3/1
Sneezing		1/0	1/0				2/0
Sore throat			1/0	1/1			2/1
Missing reports		1/0	3/0	1/0			5/0

All individuals were asymptomatic at the time of study and had a history of exposure to SARS-CoV-2. Reported symptoms occurred within the previous three weeks

In 39 of the 76 participants (51%), PCR of the combined oro-nasopharyngeal swab confirmed SARS-CoV-2 infection. In contrast, SARS-CoV-2 was detected in 13 mouthwashes (17%) of which all were positive by the oro-nasopharyngeal swab (Table 2). When considering the oro-nasopharyngeal swab as gold standard, the sensitivity of mouthwash was 33%.

**Table 2:** Comparison of mouthwash and combined oro-nasopharyngeal swab in detecting SARS-CoV-2

	Oro-nasopharyngeal swab positive	Oro-nasopharyngeal swab negative	Total
Mouthwash positive	13	0	13
Mouthwash negative	26	37	63
Total	39	37	76

The sensitivity of mouthwash is 33%, the specificity 100% when using the combined oro-nasopharyngeal swab as gold standard (McNemar test *p *value < 0.001)

**Fig. 1:**
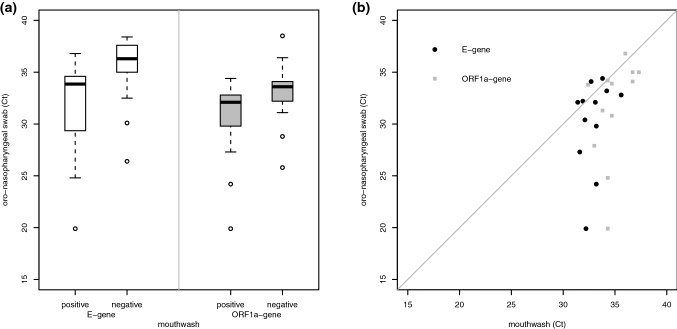
The combined oro-nasopharyngeal swab has a higher sensitivity than mouthwash. **a** Cycle threshold (*Ct*) values for *Orf1/a*- and *E*-gene for oro-nasopharyngeal swabs in samples positive and negative in mouthwash. A lower *Ct*-value indicates a higher viral load. *Ct*-values (mean, standard deviation [range]): for *E*-gene positive samples 31.46, 4.96 [19.9–36.8]; for *E*-gene negative samples 35.67, 2.71 [26.4–38.4]; *Orf1/a*-gene positive samples 30.37, 4.23 [19.9–34.4]; *Orf1/a*-gene negative samples 33.17, 2.90 [25.8–38.5], respectively. Mann–Whitney *U*-test *E*-gene: *p *value < 0.001, *Orf1/a*-gene: *p *value = 0.036. **b** *Ct*-values for samples positive in both specimen types. *Ct*-values for the mouthwash (mean, standard deviation [range]: *E*-gene 34.85, 1.53 [32.4–37.3]; *Orf1/a*-gene 32.9, 1.2 [31.4–35.6]) were higher than for the combined oro-nasopharyngeal swabs (*E*-gene 31.46, 4.96 [19.9–36.8]; *Orf1/a*-gene 30.21, 4.37 [19.9–34.4]), indicating a lower viral load in mouthwash. Only 12 paired samples were shown, since one sample was positive in the *E*-gene and another in the *Orf1/a*-gene, only (Wilcoxon signed rank test *E*-gene: *p *value = 0.007, *Orf1/a*-gene: *p *value = 0.037)

The cycle threshold (*Ct*)-value is a measure for the abundance of the transcript in the sample and correlates with viral load. When comparing *Ct*-values of oro-nasopharyngeal swabs, specimens that were positive by mouthwash had lower *Ct*-values than specimens negative by mouthwash, indicating a lower viral load in mouthwash (Fig. 1a). Samples that were positive by both methods showed higher *Ct*-values in the mouthwash (Fig. 1b). All results are consistent with a lower sensitivity of detection in mouthwash.

## Discussion

The shortage of swabs that are suitable for PCR diagnostics and the unpleasant experience frequently reported with oro-nasopharyngeal swabs, in particular in children, led us to explore the utility of mouthwash in a controlled study. We found a very low sensitivity of mouthwash (33%), when using oro-nasopharyngeal swabs as comparator. We speculate that this striking difference in sensitivity is partly due to the dilution of the mouthwash sample. Thus, mouthwash is not suitable for the reliable detection of SARS-CoV-2 infection.

Only one other small study compared throat washings and swabs [9]. In this study, the rate of detection of SARS-CoV-2 was higher in self-collected throat washings with sterile normal saline than in nasopharyngeal swabs [9]. However, the small sample size of 11 patients does not allow firm conclusions.

Our study has several strengths: we conducted the study in a controlled setting with specifically trained personnel. This allows for a more rigorously sampling than in an observational study conducted in the clinical setting. As gold standard, we chose combined oro-nasopharyngeal swabs. A systematic review that assessed the positivity rate of different specimens found that nasopharyngeal swabs had a slightly higher positivity rate than oropharyngeal swabs, with larger differences when sampling was performed more than 14 days after symptom onset [6].

Our study population were asymptomatic individuals, with a previous positive PCR-test for SARS-CoV-2 and their close contacts. Since the viral load decreases over time, this population is expected to have a low viral load and thus high *Ct*-values. Indeed, 34 of 39 (87%) positive samples had *Ct*-values above 30 for the *E-*gene, a value currently discussed as a cut-off for infection. Thus, this study was designed to rigorously assess differences in sensitivity.

Our study has also limitations. Although mouthwash with gargling was conducted under supervision, we observed some variation in adherence to the protocol regarding the duration and intensity of gargling, which may have influenced the results. Furthermore, we did not compare different RNA extraction methods, which may show a better performance with mouthwash specimens.

There is a high likelihood of aerosol formation during gargling. Thus, mouthwash should be performed alone in a well-ventilated area. This may limit its use in patients to minimise exposure of health-care personnel. In conclusion, SARS-CoV-2 detection with mouthwash showed a low sensitivity compared to oro-nasopharyngeal swabs. Thus, we do recommend performing combined oro-nasopharyngeal swabs, especially in patients with no or mild symptoms.
